# Analysis of western lowland gorilla (*Gorilla gorilla gorilla*) specific *Alu* repeats

**DOI:** 10.1186/1759-8753-4-26

**Published:** 2013-11-22

**Authors:** Adam T McLain, Glenn W Carman, Mitchell L Fullerton, Thomas O Beckstrom, William Gensler, Thomas J Meyer, Christopher Faulk, Mark A Batzer

**Affiliations:** 1 Department of Biological Sciences, Louisiana State University, Baton Rouge, LA 70803, USA; 2 Department of Bioengineering, Clemson University, Clemson, SC 29634, USA; 3 Department of Behavioral Neuroscience, Oregon Health & Science University, Portland, OR 97239, USA; 4 Department of Environmental Health Sciences, University of Michigan, Ann Arbor, MI 48109, USA

**Keywords:** Primate, Gorilla, SINE, Retrotransposon, Mobile elements

## Abstract

**Background:**

Research into great ape genomes has revealed widely divergent activity levels over time for *Alu* elements. However, the diversity of this mobile element family in the genome of the western lowland gorilla has previously been uncharacterized. *Alu* elements are primate-specific short interspersed elements that have been used as phylogenetic and population genetic markers for more than two decades. *Alu* elements are present at high copy number in the genomes of all primates surveyed thus far. The *Alu*Y subfamily and its derivatives have been recognized as the evolutionarily youngest *Alu* subfamily in the Old World primate lineage.

**Results:**

Here we use a combination of computational and wet-bench laboratory methods to assess and catalog *Alu*Y subfamily activity level and composition in the western lowland gorilla genome (gorGor3.1). A total of 1,075 independent *Alu*Y insertions were identified and computationally divided into 10 subfamilies, with the largest number of gorilla-specific elements assigned to the canonical *Alu*Y subfamily.

**Conclusions:**

The retrotransposition activity level appears to be significantly lower than that seen in the human and chimpanzee lineages, while higher than that seen in orangutan genomes, indicative of differential *Alu* amplification in the western lowland gorilla lineage as compared to other Homininae.

## Background

*Alu* elements are a family of primate-specific SINEs (Short INterspersed Elements) of approximately 300 base pairs (bp) long and present in the genomes of all living primates [1-3]. *Alu* elements were derived from 7SL RNA, the RNA component of the signal recognition particle, in the common ancestor of all living primates [4]. In the past approximately 65 million years *Alu* elements have become widely distributed in primate genomes [1,5]. *Alu* elements are now present at copy numbers of >1,000,000 in all surveyed great ape genomes (Additional file 1) [1]. Despite their high copy number the majority of *Alu* elements are genomic fossils, non-propagating relics passed down over millions of years after earlier periods of replicative activity [1,6]. It is hypothesized that a relatively small number of ‘master’ elements are responsible for the continued spread of all active subfamilies [7,8].

As non-autonomous retrotransposons, *Alu* elements do not encode the enzymatic machinery necessary for self-propagation [1,2]. This is accomplished by appropriating the replication machinery [2,9] of a much larger, autonomous retrotransposon called LINE1 (L1) via a process termed target-primed reverse transcription (TPRT) [10-13].

The effective use of SINEs as phylogenetic markers was first demonstrated in 1993 in a study seeking to resolve relationships between Pacific salmonid species [14]. Subsequent to this study, SINE-based phylogenetic methods have been applied across a wide range of species to determine evolutionary relationships [15,16]. In particular, *Alu* elements have proven to be extremely useful tools for elucidating evolutionary relationships between primate species [1,17]. The essentially homoplasy free presence of an *Alu* element of the same subfamily at a given locus between two or more primate species is almost always definitive evidence of shared ancestry [18]. The possibility of confounding events is very small, and easily resolved by the sequencing and examining of the element in question [1,18]. In the past 15 years *Alu*-based phylogenetic methods have been used with great success to resolve evolutionary relationships among the Tarsiers [19,20], New World [21] and Old World monkeys [22-24], gibbons [25], lemurs [26,27], and great apes [28].

In addition to phylogenetic applications *Alu* elements also function as effective markers for the study of population genetics via examination of polymorphic elements between members of the same species [2,29,30]. *Alu* elements are also linked to numerous genetic diseases, and the insertion of an element at an importune genomic location can have grave consequences for the individual involved [3,31,32]. Additionally, *Alu* elements are thought to be a causal factor in genomic instability [33-36].

*Alu* elements are classified in multiple major subfamilies and numerous smaller, derivative subfamilies based on specific sequence mutations [37-40]. All extant primates share older elements, while all primate lineages examined also have younger, lineage-specific subfamilies [41]. *Alu* subfamily evolution is parallel, not linear, and various subfamilies have been found to be actively retrotransposing at the same time in all primate genomes surveyed; each primate lineage thus possesses its own *Alu* subfamilies [1,42,43].

The *Alu*J subfamily is the most ancient *Alu* lineage, and was largely active from approximately 65 million years ago to approximately 55 million years ago, at which point *Alu*S evolved and supplanted *Alu*J as the predominant active subfamily [37,41]. Due to the antiquity of the lineage, *Alu*J subfamilies are present in all extant primates, including Strepsirrhines [27,44]. *Alu*S, on the other hand, evolved from *Alu*J after the Strepsirrhine-Haplorrhine divergence, and so is only found in New World and Old World primates [2,37,45]. The *Alu*Y subfamily subsequently evolved from *Alu*S in the Old World primate lineage, and remains the predominant active subfamily in catarrhines [1,41,45].

A number of *Alu*Y-derived subfamilies continue to be active in great apes [1], and polymorphic lineage-specific *Alu* elements have been well documented between existing human populations [2], indicating a continued activity level for these mobile elements. A rate of one new element in every approximately 20 live births has been proposed as the current rate of *Alu* element activity in the extant human population, but the large size of this population coupled with human generation time would make it very difficult for new elements to come to fixation outside of small population groups [46,47]. Research into *Alu* element activity in Sumatran and Bornean orangutans has indicated a comparatively low-level of continued retrotransposition activity in these apes [48], suggesting some alteration of the propagation of *Alu* within this lineage [49].

The western lowland gorilla (*Gorilla gorilla gorilla*), a subspecies of the western gorilla (*Gorilla gorilla*), is a critically endangered great ape endemic to the forests and lowland swamps of central Africa [50,51]. Western lowland gorillas are gregarious, living in family groups comprised of a dominant male, multiple females, subadult males, and juvenile offspring [52]. Western lowland gorillas are in danger of extinction due to human activity. Their wild population size is shrinking in the face of anthropogenic pressure and diseases such as Ebola [50]. Gorillas are a close evolutionary relative of humans and the *Pan* lineage of chimpanzees and bonobos, with the most widely accepted date for a common ancestor 6 to 9 million years ago [28,53-55], though a date as early as 10 million years ago has been recently proposed [56].

The genome of ‘Kamilah’ , a female western lowland gorilla living at the San Diego Zoo, was initially assembled from 5.4 Gbp of capillary sequence and 166.8 Gbp of Illumina read pairs, and further refined using bacterial artificial chromosome (BAC) and fosmid end pair capillary technology [57]. This sequence is available from the Wellcome Trust-Sanger Institute.

Previous analyses of *Alu* elements in gorillas have been limited to analysis in the context of wider research projects [28,58-61] and have not focused specifically on subfamily analysis. Here we examine the western lowland gorilla genome (build gorGor3.1) [57] to identify gorilla-specific *Alu*Y subfamilies and assess the activity levels, copy number, and age of these subfamilies. Our final analysis resulted in the identification of 1,075 *Gorilla* specific *Alu* element insertions.

## Results and discussion

### Computational examination of the western lowland gorilla genome

A total of 1,085,174 *Alu* elements were identified in the genome of the western lowland gorilla (Additional file 1). Of these, 286,801 were identified as belonging to the ancient *Alu*J subfamily, and 599,237 were identified as members of the *Alu*S subfamily. A total of 57,427 elements were too degraded or incompletely sequenced to be assigned a subfamily designation by RepeatMasker, and were simply identified as ‘*Alu*’. We identified 141,709 members of the *Alu*Y subfamily. This subfamily is of particular interest due to its relatively young age and known continued mobility in other great ape genomes [1,62]. Approximately one-third (57,458) of these putative *Alu*Y elements were >250 bp in length. Gorilla-specific elements were subsequently identified by comparison of orthologous loci in the genomes of human, common chimpanzee, and orangutan [63]. Putative unique, gorilla-specific *Alu*Y insertions were estimated at 4,127 copies. This number is similar (96.5%) to the 4,274 gorilla-specific *Alu* elements identified using other approaches [58]. Individual examination demonstrated that the majority of our 4,127 loci were in fact shared insertions. These loci were manually examined for gorilla specificity using BLAT [64]. This manual examination excluded 2,858 loci from further analysis due to the presence of shared insertions missed by Lift Over (2,626 insertions) or the lack of orthologous flanking regions in the genomes of other species that preclude PCR verification (232 insertions). This resulted in a total of 1,269 likely gorilla-specific *Alu* insertion loci for inclusion in subfamily structure analysis.

**Figure 1:**
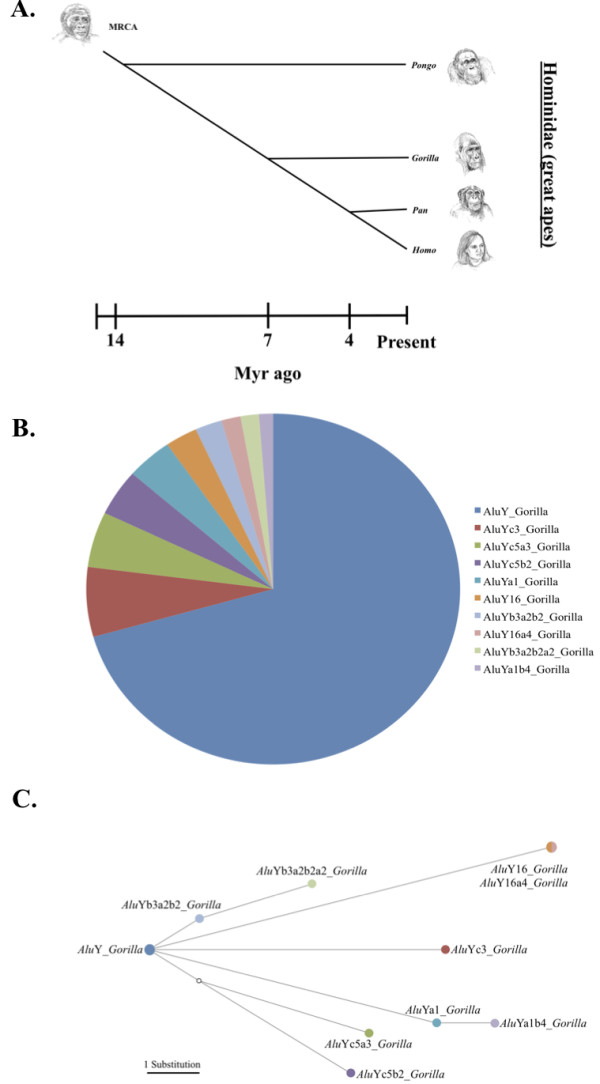
**Analysis of gorilla-specific *Alu *subfamilies. (A)** A schematic diagram of a tree of evolutionary relationships of the four genera in Family Hominidae (great apes) based on divergence dates of 6 to 9 million years ago for the *Gorilla*-*Homo/Pan* speciation event [28,53-55]. **(B)** A pie chart showing a color-coded distribution of Gorilla-specific *Alu*Y subfamilies. *Alu*Y_*Gorilla* is the largest subfamily, representing slightly less than three-fourths of the total copy number identified. **(C)** A stepwise analysis of the relationships between Gorilla-specific *Alu*Y subfamilies generated from a Network analysis of the consensus sequences for each subfamily. The color of the dots representing each subfamily are correlated with the colors in the pie chart in Figure 1B.

**Figure 2:**
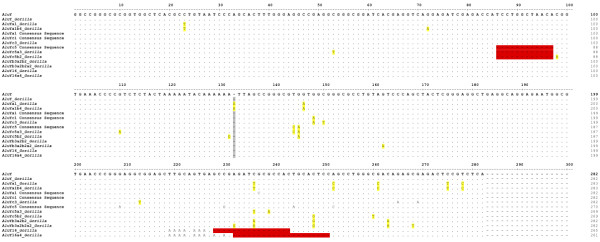
***Alu *sequence alignment.** The consensus sequence for the *Alu*Y subfamily is shown at the top, with western lowland gorilla-specific *Alu* subfamilies listed below. The dots below the consensus denote the same base with insertions and deletions noted by dashes and mutations with the appropriate bases. The consensus sequences for the *Alu*Ya1, *Alu*Yc1, and *Alu*Yc5 subfamilies included for comparative purposes. Subfamily-specific diagnostic mutations are highlighted in yellow. Lineage-specific deletions are highlighted in red. *Alu*Y_*Gorilla* is 100% identical to the *Alu*Y consensus sequence. The shared 12-bp deletion identifying the *Alu*Yc5-derived *Gorilla* subfamilies is located at position 86. The 16-bp and 20-bp deletions identifying the *Alu*Y16_*Gorilla* and *Alu*Y16a4_*Gorilla* subfamilies are visible at positions 228 and 232.

These 1,269 loci were analyzed for subfamily structure using the COSEG program. COSEG removed 194 probable gorilla-specific *Alu* insertions from the dataset due to the presence of truncations or deletions in diagnostic regions of the element, leaving 1,075 probable gorilla-specific *Alu* insertion loci for further analysis Additional file 2. COSEG then divided the loci into 10 subfamilies based on diagnostic mutations in the sequence of the individual *Alu* elements and provided subfamily consensus sequences (Figure 1) [43]. The consensus sequences were then aligned with known human *Alu*Y subfamilies from the RepBase database of repetitive elements [65] (Figure 2). A gorilla-specific nomenclature system was created to designate subfamilies using the suffix ‘*Gorilla*’ preceded by the subfamily affiliation based on a comparison to identified human subfamilies (for example, ‘*Alu*Yc5a1_*Gorilla*’). Subfamilies were named in accordance with established practice for *Alu* subfamily nomenclature [41]. The first identified *Alu*Yc5-derived subfamily was, for example, designated *Alu*Yc5a3_*Gorilla*. The ‘a’ denotes the fact that this is the first Yc5-derived subfamily identified. The ‘3’ denotes the number of diagnostic mutations by which this gorilla-specific subfamily differs from the human *Alu*Yc5 consensus sequence [41]. Subfamily age estimates were calculated using the BEAST (Bayesian Evolutionary Analysis by Sampling Trees) program [66]. 

### *Alu*Y subfamily activity in the western lowland gorilla genome

Computational and PCR analysis of the western lowland gorilla genome has identified 1,075 independent, gorilla-specific *Alu*Y insertion loci. Computational analysis of this dataset indicates the presence of 10 distinct subfamilies identifiable by the presence of diagnostic mutations specific to each lineage. The 1,075 elements identified in our study almost certainly do not represent the total number of *Alu*Y specific to western lowland gorilla genome. Any loci under our arbitrary length of >250 were excluded from our dataset. It is also likely that a number of *Alu*Y loci are located in portions of the genome where sequence data is incomplete; within repeat regions, for example. Additionally, some *Alu*Y loci were excluded when no orthologous genomic region was present in the species being used for comparison.

The largest newly identified gorilla-specific *Alu* subfamily was designated as *Alu*Y_*Gorilla*. This designation was established via computational evaluation and manual alignment of the 759 elements assigned to this subfamily. The consensus sequence for these elements was found to be 100% identical to the canonical *Alu*Y human consensus sequence (Figure 2). This subset of classic *Alu*Y elements continued to propagate in the *Gorilla* lineage after the divergence from the shared common ancestor with the *Homo*-*Pan* lineage. We assayed and verified a total of 135 loci from this subfamily via PCR (18%). The 43 elements belonging to the *Alu*Ya1_*Gorilla* subfamily differ from the *Alu*Y consensus sequence by one diagnostic mutation at nucleotide position 133. We assayed and verified via PCR 21 elements in this subfamily (49%). This sequence should not be confused with the *Homo*-*Pan Alu*Ya subfamily.

The *Alu*Ya1b4 subfamily is derived from *Alu*Ya1_*Gorilla* and is a small and very likely young subfamily of 13 elements that shared the diagnostic mutation at position 133 of Ya1 but has also accrued four additional diagnostic mutations. We assayed and verified via PCR seven elements in this subfamily (54%). A second identified *Alu*Y lineage in gorilla is the *Alu*Yc3_*Gorilla* subfamily. We assayed and verified via PCR 20 of the 69 elements in this subfamily (29%). The consensus sequence for the 69 members identified in this subfamily is a 100% match to the human *Alu*Yc3 subfamily consensus sequence (Figure 2).

Two additional gorilla-specific *Alu*Yc-derived subfamilies share the characteristic 12 bp deletion at positions 87–98 that is a hallmark of human *Alu*Yc5. These two subfamilies possess independent diagnostic mutations that make them distinct from the *Alu*Yc5 consensus sequence. These two subfamilies are designated as *Alu*Yc5a3_*Gorilla* (55 elements identified) and *Alu*Yc5b2_*Gorilla* (46 elements identified)*. Alu*Yc5a3*_Gorilla* has three additional diagnostic mutations differentiating it from the *Alu*Yc5 consensus as a mark of identification. In keeping with *Alu* subfamily naming convention this subfamily has thus been deemed ‘Yc5a3’, ‘a’ as the first Yc5-like subfamily identified in the gorilla genome and ‘3’ for the three diagnostic mutations differentiating it from the canonical Yc5 consensus. We assayed and verified 27 members of this subfamily via PCR (49%). *Alu*Yc5b2 also shares the characteristic 12 bp deletion of the human *Alu*Yc5, but has two independent diagnostic mutations (Figure 2). We assayed and verified via PCR 19 members of this subfamily (41%). It is probable that *Alu*Yc5a3_*Gorilla* and AluYc5b2_*Gorilla* derived from *Alu*Yc5 around the time of the *Homo*/*Pan*-*Gorilla* speciation event.

A third lineage nearly identical to human *Alu*Yb3a2 was identified as *Alu*Yb3a2b2_*Gorilla* (25 elements identified). This *Alu* subfamily contains two additional diagnostic mutations. Termed *Alu*Yb3a2b2_*Gorilla*, this lineage is an independent evolution in the *Gorilla gorilla gorilla* genome and not a derivative of the human-specific *Alu*Yb3a2. The *Alu*Yb lineage is human specific, meaning any identical or apparently derived *Alu* lineages in other primate genomes must be examples of independent evolution [67]. This is confirmed by the lack of orthologs at the same location in the human genome. We assayed and verified 14 members of this subfamily via PCR (56%). An additional subfamily present at only 17 copies and derived from *Alu*Yb3a2b2_*Gorilla* was identified and termed *Alu*Yb3a2b2a2_*Gorilla*, due to two diagnostic mutations separating these otherwise identical subfamilies. We assayed and verified via PCR nine elements in this subfamily (53%). The low copy number of these subfamilies coupled with their lack of impairing point mutations, even with the caveat that some subfamily members may have been overlooked, leads us to posit that they are among the youngest and potentially still active subfamilies in the western lowland gorilla genome.

Two additional subfamilies were identified that, while clearly *Alu*Y derived, do not follow the consensus sequences of established subfamilies available via RepBase. The first, termed *Alu*Y16_*Gorilla* is identified clearly by the presence of an A-rich insert at position 219 followed by a 16 bp deletion, and is present in 30 copies. We assayed and verified via PCR 10 members of this subfamily (33%). The second subfamily, apparently derived from the first and designated *Alu*Y16a4_*Gorilla*, is present in 18 copies and can be distinguished from *Alu*Y16_*Gorilla* by a 20 bp deletion occurring after the A-rich region at position 219. Seventeen elements from this subfamily were assayed via PCR (94%), with 100% of these 17 being verified as gorilla-specific. One locus (gorGor3.1 chrX:74544052–74544324) lacked sufficient orthologous 5′ sequence in non-gorilla outgroups to successfully design a working primer, but was included in the total count based on computational verification. The accumulation of non-diagnostic mutations in these two subfamilies may indicate that they are more ancient.

Approximately 25% of the 1,075 gorilla-specific *Alu*Y elements computationally identified in this study were verified by PCR, with the remaining approximately 75% verified by manual examination of computational data. It is important to note that we had no false positives in this study, and 100% of the elements computationally identified as gorilla-specific that were subsequently assayed via PCR were confirmed to be, in fact, gorilla-specific.

One means of identifying potential master elements [7] is to look for subfamily members with mutation-free polyA-tails [68]. A possible source element for the *Alu*Y_*Gorilla* subfamily, for instance, was identified on chrX:5135584–5135921, with a mutation-free 30 bp polyA-tail and intact promoter region. A posited source element for the *Alu*Yc5b2 subfamily was identified on chr9:17925753–17926051, also with a mutation-free 30 bp polyA-tail and intact promoter region.

*Alu*Y retrotransposition rates appear to be lower in the western lowland gorilla genome than in the human or chimpanzee genomes [69], while higher than that seen in the orangutan genome [48,49]. Factors influencing rates of retrotransposition are myriad [1,46]. Active retrotransposons are frequently polymorphic within a population, and are easily lost during events like speciation or population bottlenecks [70,71]. The number of active elements, and the amplification rate of elements surviving such an event, can vary greatly and impact overall retrotransposition activity in the host genome.

A possible explanation for this lower activity level include inhibition of retrotransposition in the *Gorilla* lineage by the interaction of host factors such as members of the APOBEC family of proteins with the enzymatic machinery of L1 [1,72]. Interference with L1 and *Alu* retrotransposition by APOBEC has been documented [72-74]. Analysis of the activity level of *Gorilla*-specific L1 elements could elucidate this, but has not yet been done. Additionally, environmental stress factors may impact retrotransposition rates [75]. It is possible that one or a combination of these retrotransposition-inhibiting factors could be responsible for the lower level of *Alu*Y activity in the western lowland gorilla genome.

A median joining tree of relationships between gorilla-specific *Alu*Y subfamilies was generated from a stepwise alignment [76] using the Network program (Figure 1) [42,77]. The tree generated supports the divergence of all gorilla-specific subfamilies from the *Alu*Y_*Gorilla* subfamily, and analysis of subfamily ages using BEAST places the date for this subfamily divergence at the stem of the *Gorilla* lineage. Initial divergence of gorilla-specific subfamilies occurred shortly after the speciation event separating the *Gorilla* lineage from the *Homo*-*Pan* lineage 6 to 9 million years ago [28,53-55], and master elements have continued to produce copies of each subfamily at varying rates since [7].

### Divergence dates of gorilla-specific *Alu*Y subfamilies

BEAST analysis of individual subfamily ages suggests no delay or change in transposon activity in western lowland gorilla following the divergence of the *Gorilla* and *Homo*-*Pan* lineages. The age of the gorilla-specific lineages ranges from 6.5-6.71 million years ago based on a baseline divergence of 7 million years ago for the most recent common ancestor of *Gorilla* and *Homo*-*Pan*. This indicates that all of the identified subfamilies originated around the time of the speciation event that separated these two lineages*.* This result is consistent with the ongoing propagation of these subfamilies before, during, and after the speciation event at a relatively constant rate. This indicates that the ‘master genes’ [7] from which these subfamilies are derived already existed and were retrotranspositionally active prior to the aforementioned speciation event, and have remained active subsequently. Examination of *Alu* elements indicates retrotranspositionally active elements are relatively rare, and that most *Alu* activity is the result of a small number of ‘master’ copies engaging in retrotranspositional activity over time [7]. Our results suggest that the 10 gorilla-specific *Alu*Y subfamilies identified in this study diverged and are still diverging from master elements already present in the genome of the common ancestor of the *Gorilla* and *Homo*-*Pan* lineages. A table listing each subfamily, the ‘master gene’ or ancestral *Alu* subfamily from which it was likely derived, the % divergence from the consensus sequence of the master element, copy number, and suggested age of the most recent common ancestral element are available in the Additional files section of this paper as Additional file 3.

## Conclusions

*Alu*Y subfamily activity appears to be greatly reduced in the western lowland gorilla genome when compared to the human and chimpanzee genomes. The level of activity seen, while not as low as that observed in the genome of the orangutan, is consistent with a change in host surveillance or intrinsic retrotransposition capacity. *Alu* subfamily age estimates provide further support for the master gene model of *Alu* retrotransposition with a relatively low number of ancient lineages responsible for ongoing retrotranspositional activity. The 1,075 lineage specific *Alu*Y insertion loci and the 10 subfamilies identified should provide future researchers with a rich source of genetic systems for conservation biology and evolutionary genetics.

## Methods

### Computational methodology

The genome of the Western lowland gorilla (*Gorilla gorilla gorilla*) was downloaded and analyzed for the presence of *Alu* elements using an in-house installation of the RepeatMasker program [62]. The *Gorilla gorilla gorilla* genome is available for download and analysis via the website of the Wellcome Trust-Sanger Institute [78]. The resulting dataset was parsed into separate files based on the *Alu* subfamily designations assigned by RepeatMasker. The file containing elements designated as members of the *Alu*Y subfamily was then further parsed to remove 84,251 elements under the length of 250 bp using the estimation that shorter elements were likely to be older elements present in multiple species and therefore not useful for our analysis. The ‘Fetch Sequences’ function from the online version of the Galaxy suite of programs [63,79-81] was then used to retrieve the individual DNA sequence present at each of these loci using the gorilla genome build gorGor3.1, and the Lift Over function was used to examine these loci for gorilla lineage specificity by comparison to the closely related genomes of human (*Homo sapiens*; hg19), chimpanzee (*Pan troglodytes*; panTro2), and Sumatran orangutan (*Pongo pygmaeus abelii*; ponAbe2). An additional 200 bp of flanking sequence on each side of the loci assayed was included in this analysis for validation of orthologous loci between the nine primate species considered in this study (Table 1).

**Table 1:** DNA sample data of all species examined in this study

**Taxonomic name**	**Common name**	**Origin**	**ID number**
*Gorilla gorilla gorilla*	Western lowland gorilla	Coriell^1^	AG05251
*Homo sapiens*	Human, HeLa	ATCC^2^	HeLa CCL-2
*Pan troglodytes*	Common chimpanzee	IPBIR^3^	NS06006
*Pan paniscus*	Bonobo	IPBIR^3^	PR00661
*Pongo pygmaeus*	Bornean orangutan	Coriell^1^	AG05252A
*Pongo abelii*	Sumartran orangutan	Coriell^1^	GM06213A
*Nomascus leucogenys*	Northern white-cheeked gibbon	Carbone Lab^4^	NLL606
*Macaca mulatta*	Rhesus macaque	Coriell^1^	NG07098
*Chlorocebus aethiops*	African green monkey	ATCC^2^	CCL70

^1^Coriell Institute for Medical Research, 403 Haddon Avenue, Camden, NJ 08103, USA.

^2^From cell lines provided by American Type Culture Collection (ATCC), P.O. Box 1549, Manassas, VA 20108, USA.

^3^Integrated Primate Biomaterials and Information Resource (IPBIR), http://ccr.coriell.org/Sections/Collections/.

^4^Laboratory of Dr. Lucia Carbone, Oregon Health & Science University, Beaverton, Oregon, http://carbonelab.com/.

Loci selected for verification were examined for further evidence of gorilla-specificity using the BLAST-Like Alignment Tool (BLAT) available at the UCSC Genome Browser website [82]. Putative gorilla-specific loci were compared to the available genomes of three other primate species, human (hg19), chimpanzee (panTro2), and orangutan (ponAbe2) [64,83]. Elements found to be absent in these species and with sufficient orthologous flanking across species were marked for PCR primer design and experimental validation. Loci determined to be shared insertions, as well as those lacking sufficient orthologous flanking for effective primer design, were removed from further consideration [64].

The COSEG program [84], designed to identify repeat subfamilies using significant co-segregating mutations, was then run on the remaining putative gorilla-specific insertions to identify and group specific subfamilies together. COSEG ignores non-diagnostic mutations during analysis, providing an accurate representation of relationships between subfamilies of elements by ignoring potentially misleading mutational events [43]. COSEG uses a minimum subfamily size of 50 elements as the default setting. We arbitrarily defined subfamilies as groups of >10 elements to increase the detail of subfamily structure resolved. A subset of a minimum of 10% of each identified subfamily was then chosen for verification using locus-specific PCR, with a total of 279 loci assayed and verified (Figure 1).

A multi-species alignment comprised of the species listed above was created for each locus using BioEdit [85]. Oligonucleotide primers for the PCR assays were designed in shared regions flanking each putative gorilla-specific element chosen for experimental verification using the Primer3Plus program [86]. These primers were then tested computationally against available primate genomes using the *in-silico* PCR tool on the UCSC Genome Bioinformatics website [83].

Subfamily age estimates were calculated using the BEAST program [66,87]. BEAST has previously been used to estimate dates of divergence using transposon data [88]. For each subclade, the consensus sequence for each subfamily was determined from the COSEG output [43]. The progenitor element was determined by RepeatMasker analysis of each consensus sequence. Elements were aligned using the SeaView software program and MUSCLE algorithm [76,89]. The progenitor element was then used as an out-group to root the tree of each subclade. BEAST was calibrated with a baseline divergence date of 7 million years ago for the split between the *Gorilla* and *Homo*-*Pan* lineages. A divergence date of 7 million years ago is within the generally accepted 6 to 9 million years ago range for this divergence [28,53-55]. BEAST was run with the following parameters: Site Heterogeneity = ‘gamma’; Clock = ‘strict clock’; Species Tree Prior = ‘birth death process’; Prior for Time of Most Recent Common Ancestor (tmrca) = ‘Normal distribution’ with mean of 7.0 million years and 1.0 standard deviation’; ucld.mean = ‘uniform model’ with initial rate set at 0.033; Length of Chain = ‘10,000,000’; all other parameters were left at default settings.

The Network program [90] was run on gorilla-specific *Alu*Y subfamily consensus sequences to generate a stepwise tree of relationships between identified subfamilies [42,77]. The consensus sequences for the gorilla-specific *Alu*Y subfamilies were aligned using the MUSCLE algorithm [76] and converted to the .rdf file format using the DNAsp program [91]. The .rdf file was then imported to Network, and a median-joining analysis was run. The resulting output file demonstrating evolutionary relationships between subfamilies is presented in Figure 1C.

### PCR and DNA sequencing

To verify gorilla-specificity, locus specific PCR was performed with a nine-species primate panel comprised of DNA samples from the following species: Western lowland gorilla (*Gorilla gorilla gorilla*); Human HeLa (*Homo sapiens*); Common chimpanzee (*Pan troglodytes*); Bonobo (*Pan paniscus*); Bornean orangutan (*Pongo pygmaeus*); Sumatran orangutan (*Pongo abelii*); Northern white-cheeked gibbon (*Nomascus leucogenys*); Rhesus macaque (*Macaca mulatta*); African green monkey (*Chlorocebus aethiops*). Information on all DNA samples used in PCR analysis is listed in Table 1.

**Figure 3:**
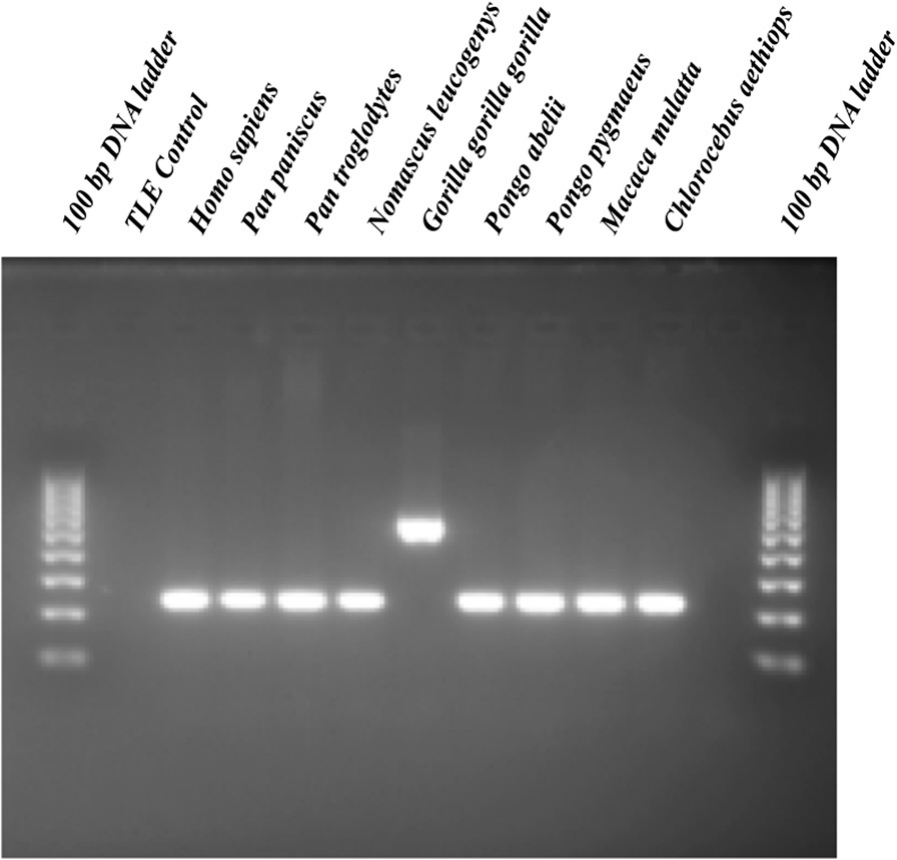
**Phylogenetic assay of a western lowland gorilla-specific *Alu *insertion (Primer Pair Gor112).** An agarose gel chromatograph of the gorilla specific *Alu* insertion Gor112. The filled site is approximately 550 bp (lane 7) and the empty site is 250 bp (lanes 3 to 6 and 8 to 11). Lanes (1) 100 bp DNA ladder; (2) negative control; (3) human; (4) bonobo; (5) common chimpanzee; (6) northern white-cheeked gibbon; (7) western lowland gorilla; (8) Sumatran orangutan; (9) Bornean orangutan; (10) Rhesus macaque; (11) green monkey; (12) empty; (13) 100 bp DNA ladder.

PCR amplification of each locus was performed in 25 μL reactions using 15 ng of template DNA, 200 nM of each primer, 200 μM dNTPs in 50 mM KCl, 1.5 mM MgCl_2_, 10 mM Tris–HCl (pH 8.4), and 2 units of *Taq* DNA polymerase. PCR reaction conditions were as follows: an initial denaturation at 95°C for 1 min, followed by 32 cycles of denaturation at 95°C, annealing at the previously determined optimal annealing temperature (60°C with some exceptions), and extension at 72°C for 30 s each, followed by a final extension of 72°C for 1 min. PCR products were analyzed to confirm gorilla specificity of all loci on 2% agarose gels stained with 0.25 ug ethidium bromide and visualized with UV fluorescence (Figure 3). A list of all 279 assayed loci, corresponding primer pairs, and optimal annealing temperatures for each are available as Additional file 4 in the Additional files for this study. Additionally, all PCR tested loci containing unidentified bases in the original sequence data were subjected to chain-termination sequencing to verify bp composition [92]. Sequence data generated from this project for gorilla-specific *Alu*Y subfamilies has been deposited in GenBank under the accession numbers (KF668269-KF668278).

## Abbreviations

BAC: Bacterial artificial chromosome; BEAST: Bayesian evolutionary analysis sampling trees; BLAST: Basic local alignment search tool; BLAT: Blast-like alignment tool; bp: Base pair; Chr: Chromosome; Gpb: Gigabase-pair; LINE: Long interspersed element; PCR: Polymerase chain reaction; SINE: Short interspersed element; TPRT: Target-primed reverse transcription; TSD: Target-site duplication; UCSC: University of California Santa Cruz.

## Competing interests

The authors declare that they have no competing interests.

## Authors’ contributions

ATM and MAB designed the research and wrote the paper. ATM, GWC, MLF, TOB, and WG performed the experiments. ATM, GWC, MLF, TOB, and WG designed the PCR primers. ATM, CF, and TJM performed the computational analyses. All authors read and approved the final manuscript.

## Supplementary Material

Additional file 1 **Enumeration of *Alu *elements in ape genomes.** The RepeatMasker program was run on the ape genomes currently available for download via Genbank. An in-house Perl script was then used to tally *Alu* elements by total copy number, and total copy number per each of the three major subfamilies. Click here for file

Additional file 2 **A complete listing of all 1,075 verified gorilla-specific *Alu *** **Y insertions.** Click here for file

Additional file 3 **Estimated age of gorilla-specific *Alu *subfamilies based on BEAST analysis.** The BEAST program was run on each gorilla-specific subfamily with a baseline divergence age of 7 million years ago to determine the age of the subfamilies, the most likely progenitor or ancestral element, and the % divergence from the consensus sequence of the ancestral subfamily. Click here for file

Additional file 4 All primer pairs used in this study listed with chromosomal location of the locus assayed and optimal annealing temperature. Click here for file

## References

[B1] Konkel MK Walker JA Batzer MA LINEs and SINEs of primate evolution Evol Anthropol 2010 19 236 249 10.1002/evan.20283 25147443 PMC4137791

[B2] Batzer MA Deininger PL Alu repeats and human genomic diversity Nat Rev Genet 2002 3 370 379 10.1038/nrg798 11988762

[B3] Cordaux R Batzer MA The impact of retrotransposons on human genome evolution Nat Rev Genet 2009 10 691 703 10.1038/nrg2640 19763152 PMC2884099

[B4] Ullu E Tschudi C Alu sequences are processed 7SL RNA genes Nature 1984 312 171 172 10.1038/312171a0 6209580

[B5] Roy-Engel AM Batzer MA Deininger PL Evolution of human retrosequences: Alu, Encyclopedia of Life Sciences 2008 Chichester: John Wiley & Sons, Ltd

[B6] Cordaux R Lee J Dinoso L Batzer MA Recently integrated Alu retrotransposons are essentially neutral residents of the human genome Gene 2006 373 138 144 16527433 10.1016/j.gene.2006.01.020

[B7] Deininger PL Batzer MA Hutchison CA 3rd Edgell MH Master genes in mammalian repetitive DNA amplification Trends Genet 1992 8 307 311 1365396 10.1016/0168-9525(92)90262-3

[B8] Han K Xing J Wang H Hedges DJ Garber RK Cordaux R Batzer MA Under the genomic radar: the stealth model of Alu amplification Genome Res 2005 15 655 664 10.1101/gr.3492605 15867427 PMC1088293

[B9] Dewannieux M Esnault C Heidmann T LINE-mediated retrotransposition of marked Alu sequences Nat Genet 2003 35 41 48 10.1038/ng1223 12897783

[B10] Luan DD Korman MH Jakubczak JL Eickbush TH Reverse transcription of R2Bm RNA is primed by a nick at the chromosomal target site: a mechanism for non-LTR retrotransposition Cell 1993 72 595 605 10.1016/0092-8674(93)90078-5 7679954

[B11] Luan DD Eickbush TH RNA template requirements for target DNA-primed reverse transcription by the R2 retrotransposable element Mol Cell Biol 1995 15 3882 3891 7540721 10.1128/mcb.15.7.3882 PMC230628

[B12] Cost GJ Feng Q Jacquier A Boeke JD Human L1 element target-primed reverse transcription in vitro EMBO J 2002 21 5899 5910 10.1093/emboj/cdf592 12411507 PMC131089

[B13] Feng Q Moran JV Kazazian HH Jr Boeke JD Human L1 retrotransposon encodes a conserved endonuclease required for retrotransposition Cell 1996 87 905 916 10.1016/S0092-8674(00)81997-2 8945517

[B14] Murata S Takasaki N Saitoh M Okada N Determination of the phylogenetic relationships among pacific salmonids by using short interspersed elements (SINEs) as temporal landmarks of evolution Proc Natl Acad Sci U S A 1993 90 6995 6999 10.1073/pnas.90.15.6995 8346208 PMC47062

[B15] Shedlock AM Okada N Sine insertions: powerful tools for molecular systematics Bioessays 2000 22 148 160 10.1002/(SICI)1521-1878(200002)22:2<148::AID-BIES6>3.0.CO;2-Z 10655034

[B16] Shedlock AM Takahashi K Okada N Sines of speciation: tracking lineages with retroposons Trends Ecol Evol 2004 19 545 553 10.1016/j.tree.2004.08.002 16701320

[B17] Minghetti PP Dugaiczyk A The emergence of new DNA repeats and the divergence of primates Proc Natl Acad Sci U S A 1993 90 1872 1876 10.1073/pnas.90.5.1872 8446601 PMC45982

[B18] Ray DA Xing J Salem AH Batzer MA SINEs of a nearly perfect character Syst Biol 2006 55 928 935 10.1080/10635150600865419 17345674

[B19] Zietkiewicz E Richer C Labuda D Phylogenetic affinities of tarsier in the context of primate Alu repeats Mol Phylogenet Evol 1999 11 77 83 10.1006/mpev.1998.0564 10082612

[B20] Schmitz J Ohme M Zischler H SINE insertions in cladistic analyses and the phylogenetic affiliations of tarsius bancanus to other primates Genetics 2001 157 777 784 11156996 10.1093/genetics/157.2.777 PMC1461532

[B21] Ray DA Xing J Hedges DJ Hall MA Laborde ME Anders BA White BR Stoilova N Fowlkes JD Landry KE Chemnick LG Ryder OA Batzer MA Alu insertion loci and platyrrhine primate phylogeny Mol Phylogenet Evol 2005 35 117 126 10.1016/j.ympev.2004.10.023 15737586

[B22] Xing J Wang H Han K Ray DA Huang CH Chemnick LG Stewart CB Disotell TR Ryder OA Batzer MA A mobile element based phylogeny of Old World monkeys Mol Phylogenet Evol 2005 37 872 880 10.1016/j.ympev.2005.04.015 15936216

[B23] Xing J Wang H Zhang Y Ray DA Tosi AJ Disotell TR Batzer MA A mobile element-based evolutionary history of guenons (tribe cercopithecini) BMC Biol 2007 5 5 10.1186/1741-7007-5-5 17266768 PMC1797000

[B24] Li J Han K Xing J Kim HS Rogers J Ryder OA Disotell T Yue B Batzer MA Phylogeny of the macaques (cercopithecidae: macaca) based on Alu elements Gene 2009 448 242 249 10.1016/j.gene.2009.05.013 19497354 PMC2783879

[B25] Meyer TJ McLain AT Oldenburg JM Faulk C Bourgeois MG Conlin EM Mootnick AR De Jong PJ Roos C Carbone L Batzer MA An Alu-based phylogeny of gibbons (hylobatidae) Mol Biol Evol 2012 29 3441 3450 10.1093/molbev/mss149 22683814 PMC3472497

[B26] McLain AT Meyer TJ Faulk C Herke SW Oldenburg JM Bourgeois MG Abshire CF Roos C Batzer MA An alu-based phylogeny of lemurs (infraorder: lemuriformes) PLoS One 2012 7 e44035 10.1371/journal.pone.0044035 22937148 PMC3429421

[B27] Roos C Schmitz J Zischler H Primate jumping genes elucidate strepsirrhine phylogeny Proc Natl Acad Sci U S A 2004 101 10650 10654 10.1073/pnas.0403852101 15249661 PMC489989

[B28] Salem AH Ray DA Xing J Callinan PA Myers JS Hedges DJ Garber RK Witherspoon DJ Jorde LB Batzer MA Alu elements and hominid phylogenetics Proc Natl Acad Sci U S A 2003 100 12787 12791 10.1073/pnas.2133766100 14561894 PMC240696

[B29] Batzer MA Stoneking M Alegria-Hartman M Bazan H Kass DH Shaikh TH Novick GE Ioannou PA Scheer WD Herrera RJ African origin of human-specific polymorphic Alu insertions Proc Natl Acad Sci U S A 1994 91 12288 12292 10.1073/pnas.91.25.12288 7991620 PMC45422

[B30] Perna NT Batzer MA Deininger PL Stoneking M Alu insertion polymorphism: a new type of marker for human population studies Hum Biol 1992 64 641 648 1328024

[B31] Deininger PL Batzer MA Alu repeats and human disease Mol Genet Metab 1999 67 183 193 10.1006/mgme.1999.2864 10381326

[B32] Hancks DC Kazazian HH Jr Active human retrotransposons: variation and disease Curr Opin Genet Dev 2012 22 191 203 10.1016/j.gde.2012.02.006 22406018 PMC3376660

[B33] Cook GW Konkel MK Major JD 3rd Walker JA Han K Batzer MA Alu pair exclusions in the human genome Mob DNA 2011 2 10 10.1186/1759-8753-2-10 21943335 PMC3215922

[B34] Hedges DJ Deininger PL Inviting instability: transposable elements, double-strand breaks, and the maintenance of genome integrity Mutat Res 2007 616 46 59 10.1016/j.mrfmmm.2006.11.021 17157332 PMC1850990

[B35] Konkel MK Batzer MA A mobile threat to genome stability: the impact of non-LTR retrotransposons upon the human genome Semin Cancer Biol 2010 20 211 221 10.1016/j.semcancer.2010.03.001 20307669 PMC2925057

[B36] Cook GW Konkel MK Walker JA Bourgeois MG Fullerton ML Fussell JT Herbold HD Batzer MA A comparison of 100 human genes using an alu element-based instability model PLoS One 2013 8 e65188 10.1371/journal.pone.0065188 23755193 PMC3670932

[B37] Jurka J Smith T A fundamental division in the Alu family of repeated sequences Proc Natl Acad Sci U S A 1988 85 4775 4778 10.1073/pnas.85.13.4775 3387438 PMC280518

[B38] Slagel V Flemington E Traina-Dorge V Bradshaw H Deininger P Clustering and subfamily relationships of the Alu family in the human genome Mol Biol Evol 1987 4 19 29 3128713 10.1093/oxfordjournals.molbev.a040422

[B39] Willard C Nguyen HT Schmid CW Existence of at least three distinct Alu subfamilies J Mol Evol 1987 26 180 186 10.1007/BF02099850 3129565

[B40] Britten RJ Baron WF Stout DB Davidson EH Sources and evolution of human Alu repeated sequences Proc Natl Acad Sci U S A 1988 85 4770 4774 10.1073/pnas.85.13.4770 3387437 PMC280517

[B41] Batzer MA Deininger PL Hellmann-Blumberg U Jurka J Labuda D Rubin CM Schmid CW Zietkiewicz E Zuckerkandl E Standardized nomenclature for Alu repeats J Mol Evol 1996 42 3 6 10.1007/BF00163204 8576960

[B42] Cordaux R Hedges DJ Batzer MA Retrotransposition of Alu elements: how many sources? Trends Genet 2004 20 464 467 10.1016/j.tig.2004.07.012 15363897

[B43] Price AL Eskin E Pevzner PA Whole-genome analysis of Alu repeat elements reveals complex evolutionary history Genome Res 2004 14 2245 2252 10.1101/gr.2693004 15520288 PMC525682

[B44] Liu GE Alkan C Jiang L Zhao S Eichler EE Comparative analysis of Alu repeats in primate genomes Genome Res 2009 19 876 885 10.1101/gr.083972.108 19411604 PMC2675976

[B45] Kapitonov V Jurka J The age of Alu subfamilies J Mol Evol 1996 42 59 65 10.1007/BF00163212 8576965

[B46] Cordaux R Hedges DJ Herke SW Batzer MA Estimating the retrotransposition rate of human Alu elements Gene 2006 373 134 137 16522357 10.1016/j.gene.2006.01.019

[B47] Xing J Zhang Y Han K Salem AH Sen SK Huff CD Zhou Q Kirkness EF Levy S Batzer MA Jorde LB Mobile elements create structural variation: analysis of a complete human genome Genome Res 2009 19 1516 1526 10.1101/gr.091827.109 19439515 PMC2752133

[B48] Locke DP Hillier LW Warren WC Worley KC Nazareth LV Muzny DM Yang SP Wang Z Chinwalla AT Minx P Mitreva M Cook L Delahaunty KD Fronick C Schmidt H Fulton LA Fulton RS Nelson JO Magrini V Pohl C Graves TA Markovic C Cree A Dinh HH Hume J Kovar CL Fowler GR Lunter G Meader S Heger A Comparative and demographic analysis of orang-utan genomes Nature 2011 469 529 533 10.1038/nature09687 21270892 PMC3060778

[B49] Walker JA Konkel MK Ullmer B Monceaux CP Ryder OA Hubley R Smit AF Batzer MA Orangutan Alu quiescence reveals possible source element: support for ancient backseat drivers Mob DNA 2012 3 8 10.1186/1759-8753-3-8 22541534 PMC3357318

[B50] Strier KB Primate behavioral ecology 2007 3 Boston, MA: Allyn and Bacon

[B51] Fleagle JG Primate adaptation and evolution 1999 2 San Diego, CA: Academic

[B52] Fleagle JG Janson CH Reed KE Primate communities 1999 Cambridge: Cambridge University Press

[B53] Steiper ME Young NM Primate molecular divergence dates Mol Phylogenet Evol 2006 41 384 394 10.1016/j.ympev.2006.05.021 16815047

[B54] Glazko GV Nei M Estimation of divergence times for major lineages of primate species Mol Biol Evol 2003 20 424 434 10.1093/molbev/msg050 12644563

[B55] Chen FC Li WH Genomic divergences between humans and other hominoids and the effective population size of the common ancestor of humans and chimpanzees Am J Hum Genet 2001 68 444 456 10.1086/318206 11170892 PMC1235277

[B56] Langergraber KE Prufer K Rowney C Boesch C Crockford C Fawcett K Inoue E Inoue-Muruyama M Mitani JC Muller MN Robbins MM Schubert G Stoinski TS Viola B Watts D Wittig RM Wrangham RW Zuberbuhler K Paabo S Vigilant L Generation times in wild chimpanzees and gorillas suggest earlier divergence times in great ape and human evolution Proc Natl Acad Sci U S A 2012 109 15716 15721 10.1073/pnas.1211740109 22891323 PMC3465451

[B57] Scally A Dutheil JY Hillier LW Jordan GE Goodhead I Herrero J Hobolth A Lappalainen T Mailund T Marques-Bonet T McCarthy S Montgomery SH Schwalie PC Tang YA Ward MC Xue Y Yngvadottir B Alkan C Andersen LN Ayub Q Ball EV Beal K Bradley BJ Chen Y Clee CM Fitzgerald S Graves TA Gu Y Heath P Heger A Insights into hominid evolution from the gorilla genome sequence Nature 2012 483 169 175 10.1038/nature10842 22398555 PMC3303130

[B58] Ventura M Catacchio CR Alkan C Marques-Bonet T Sajjadian S Graves TA Hormozdiari F Navarro A Malig M Baker C Lee C Turner EH Chen L Kidd JM Archidiacono N Shendure J Wilson RK Eichler EE Gorilla genome structural variation reveals evolutionary parallelisms with chimpanzee Genome Res 2011 21 1640 1649 10.1101/gr.124461.111 21685127 PMC3202281

[B59] Lee J Han K Meyer TJ Kim HS Batzer MA Chromosomal inversions between human and chimpanzee lineages caused by retrotransposons PLoS One 2008 3 e4047 10.1371/journal.pone.0004047 19112500 PMC2603318

[B60] Sen SK Han K Wang J Lee J Wang H Callinan PA Dyer M Cordaux R Liang P Batzer MA Human genomic deletions mediated by recombination between Alu elements Am J Hum Genet 2006 79 41 53 10.1086/504600 16773564 PMC1474114

[B61] Hormozdiari F Konkel MK Prado-Martinez J Chiatante G Herraez IH Walker JA Nelson B Alkan C Sudmant PH Huddleston J Catacchio CR Ko A Maliq M Baker C Great Ape Genome Project, Marques-Bonet T, Ventura M, Batzer MA, Eichler EE Rates and patterns of great ape retrotransposition Proc Natl Acad Sci U S A 2013 110 13457 13462 10.1073/pnas.1310914110 23884656 PMC3746892

[B62] RepeatMasker open-3.0 [http://www.repeatmasker.org]

[B63] Giardine B Riemer C Hardison RC Burhans R Elnitski L Shah P Zhang Y Blankenberg D Albert I Taylor J Miller W Kent WJ Nekrutenko A Galaxy: a platform for interactive large-scale genome analysis Genome Res 2005 15 1451 1455 10.1101/gr.4086505 16169926 PMC1240089

[B64] Kent WJ BLAT–the BLAST-like alignment tool Genome Res 2002 12 656 664 11932250 10.1101/gr.229202 PMC187518

[B65] Jurka J Kapitonov VV Pavlicek A Klonowski P Kohany O Walichiewicz J Repbase update, a database of eukaryotic repetitive elements Cytogenet Genome Res 2005 110 462 467 10.1159/000084979 16093699

[B66] Drummond AJ Rambaut A BEAST: bayesian evolutionary analysis by sampling trees BMC Evol Biol 2007 7 214 10.1186/1471-2148-7-214 17996036 PMC2247476

[B67] Carter AB Salem AH Hedges DJ Keegan CN Kimball B Walker JA Watkins WS Jorde LB Batzer MA Genome-wide analysis of the human Alu Yb-lineage Human Genomics 2004 1 167 178 15588477 10.1186/1479-7364-1-3-167 PMC3525081

[B68] Roy-Engel AM Salem AH Oyeniran OO Deininger L Hedges DJ Kilroy GE Batzer MA Deininger PL Active Alu element “A-tails”: size does matter Genome Res 2002 12 1333 1344 10.1101/gr.384802 12213770 PMC186649

[B69] Hedges DJ Callinan PA Cordaux R Xing J Barnes E Batzer MA Differential alu mobilization and polymorphism among the human and chimpanzee lineages Genome Res 2004 14 1068 1075 10.1101/gr.2530404 15173113 PMC419785

[B70] Hedges DJ Batzer MA From the margins of the genome: mobile elements shape primate evolution Bioessays 2005 27 785 794 10.1002/bies.20268 16015599

[B71] Belancio VP Hedges DJ Deininger P Mammalian non-LTR retrotransposons: for better or worse, in sickness and in health Genome Res 2008 18 343 358 10.1101/gr.5558208 18256243

[B72] Schumann GG APOBEC3 proteins: major players in intracellular defence against LINE-1-mediated retrotransposition Biochem Soc Trans 2007 35 637 642 17511669 10.1042/BST0350637

[B73] Hulme AE Bogerd HP Cullen BR Moran JV Selective inhibition of Alu retrotransposition by APOBEC3G Gene 2007 390 199 205 10.1016/j.gene.2006.08.032 17079095 PMC2917221

[B74] Bogerd HP Wiegand HL Hulme AE Garcia-Perez JL O’Shea KS Moran JV Cullen BR Cellular inhibitors of long interspersed element 1 and Alu retrotransposition Proc Natl Acad Sci U S A 2006 103 8780 8785 10.1073/pnas.0603313103 16728505 PMC1482655

[B75] Farkash EA Kao GD Horman SR Prak ET Gamma radiation increases endonuclease-dependent L1 retrotransposition in a cultured cell assay Nucleic Acids Res 2006 34 1196 1204 10.1093/nar/gkj522 16507671 PMC1385994

[B76] Edgar RC MUSCLE: multiple sequence alignment with high accuracy and high throughput Nucleic Acids Res 2004 32 1792 1797 10.1093/nar/gkh340 15034147 PMC390337

[B77] Bandelt HJ Forster P Rohl A Median-joining networks for inferring intraspecific phylogenies Mol Biol Evol 1999 16 37 48 10.1093/oxfordjournals.molbev.a026036 10331250

[B78] Wellcome trust-sanger institute gorilla genome homepage [http://www.sanger.ac.uk/resources/downloads/gorilla/]

[B79] Blankenberg D Von Kuster G Coraor N Ananda G Lazarus R Mangan M Nekrutenko A Taylor J Galaxy: a web-based genome analysis tool for experimentalists Curr Protoc Mol Biol 2010 Chapter 19 11 21 10.1002/0471142727.mb1910s89 PMC4264107 20069535

[B80] Goecks J Nekrutenko A Taylor J Galaxy: a comprehensive approach for supporting accessible, reproducible, and transparent computational research in the life sciences Genome Biol 2010 11 R86 10.1186/gb-2010-11-8-r86 20738864 PMC2945788

[B81] Galaxy [http://galaxyproject.org]

[B82] UCSC genome browser [http://genome.ucsc.edu]

[B83] Kent WJ Sugnet CW Furey TS Roskin KM Pringle TH Zahler AM Haussler D The human genome browser at UCSC Genome Res 2002 12 996 1006 12045153 10.1101/gr.229102 PMC186604

[B84] COSEG [http://www.repeatmasker.org:COSEGDownload.html]

[B85] Hall TA BioEdit: a user-friendly biological sequence alignment editor and analysis program for windows 95/98/NT Nucleic Acids Symp Ser 1999 41 95 98

[B86] Untergasser A Nijveen H Rao X Bisseling T Geurts R Leunissen JA Primer3Plus, an enhanced web interface to Primer3 Nucleic Acids Res 2007 35 W71 74 10.1093/nar/gkm306 17485472 PMC1933133

[B87] BEAST [http://beast.bio.ed.ac.uk]

[B88] Hellen EH Brookfield JF The diversity of class II transposable elements in mammalian genomes has arisen from ancestral phylogenetic splits during ancient waves of proliferation through the genome Mol Biol Evol 2013 30 100 108 10.1093/molbev/mss206 22923465 PMC3525145

[B89] Gouy M Guindon S Gascuel O SeaView version 4: a multiplatform graphical user interface for sequence alignment and phylogenetic tree building Mol Biol Evol 2010 27 221 224 10.1093/molbev/msp259 19854763

[B90] NETWORK [http://www.fluxus-engineering.com/sharenet.htm]

[B91] Librado P Rozas J DnaSP v5: a software for comprehensive analysis of DNA polymorphism data Bioinformatics 2009 25 1451 1452 10.1093/bioinformatics/btp187 19346325

[B92] Sanger F Air GM Barrell BG Brown NL Coulson AR Fiddes CA Hutchison CA Slocombe PM Smith M Nucleotide sequence of bacteriophage phi X174 DNA Nature 1977 265 687 695 10.1038/265687a0 870828

